# Antibody-Based Biologics for CNS Disorders

**DOI:** 10.3390/antib15050086

**Published:** 2026-09-16

**Authors:** Eric Ka-Wai Hui, Ruben J. Boado

**Affiliations:** 1 Department of Research and Development, Thermo Fisher Scientific, West Hills, CA 91304, USA; 2 Department of Medicine, University of California, Los Angeles (UCLA), Los Angeles, CA 90095, USA

**Keywords:** Alzheimer’s disease, bispecific antibodies, blood–brain barrier, decoy receptors, fusion proteins, insulin receptor, insulin-like growth factor 1 receptor, lysosomal storage disorders, monoclonal antibody, neurotrophins, Parkinson’s disease, protein-based therapy, receptor-mediated transcytosis, transferrin receptor

## Abstract

The blood-brain barrier (BBB) is permeable only to small lipophilic molecules, whereas hydrophilic nutrients, such as glucose and amino acids, cross the BBB via carrier-mediated transport systems. In contrast, circulating proteins do not cross the BBB. However, the BBB expresses receptors able to induce receptor-mediated transcytosis (RMT) of selective proteins across the BBB, as in the case of insulin, transferrin, and insulin-like growth factor 1 (IGF1). This led to the hypothesis that the transport of protein-based biologics to the brain may be possible by targeting BBB receptors that induce RMT. Monoclonal antibodies targeting these receptors penetrate the BBB via RMT and are distributed throughout the brain, acting as molecular Trojan horses and/or shuttle systems. A generation of brain-penetrating fusion proteins was produced targeting BBB insulin, transferrin, and IGF1 receptors, respectively, with full antibodies and/or antibody fragments acting as transport domains. These antibody-based fusion proteins were validated in various experimental models, including lysosomal storage disorders (LSDs), stroke, Parkinson’s disease, and Alzheimer’s disease (AD), respectively. Clinical trials with brain-penetrating antibody-based biologicals targeting BBB transferrin and insulin receptors have been completed or are in progress in LSDs and in AD. The aim of this review is to examine the progress made in antibody-based biologicals for CNS disorders from genetic engineering to clinical trials.

## 1. Introduction

**Figure 1:**
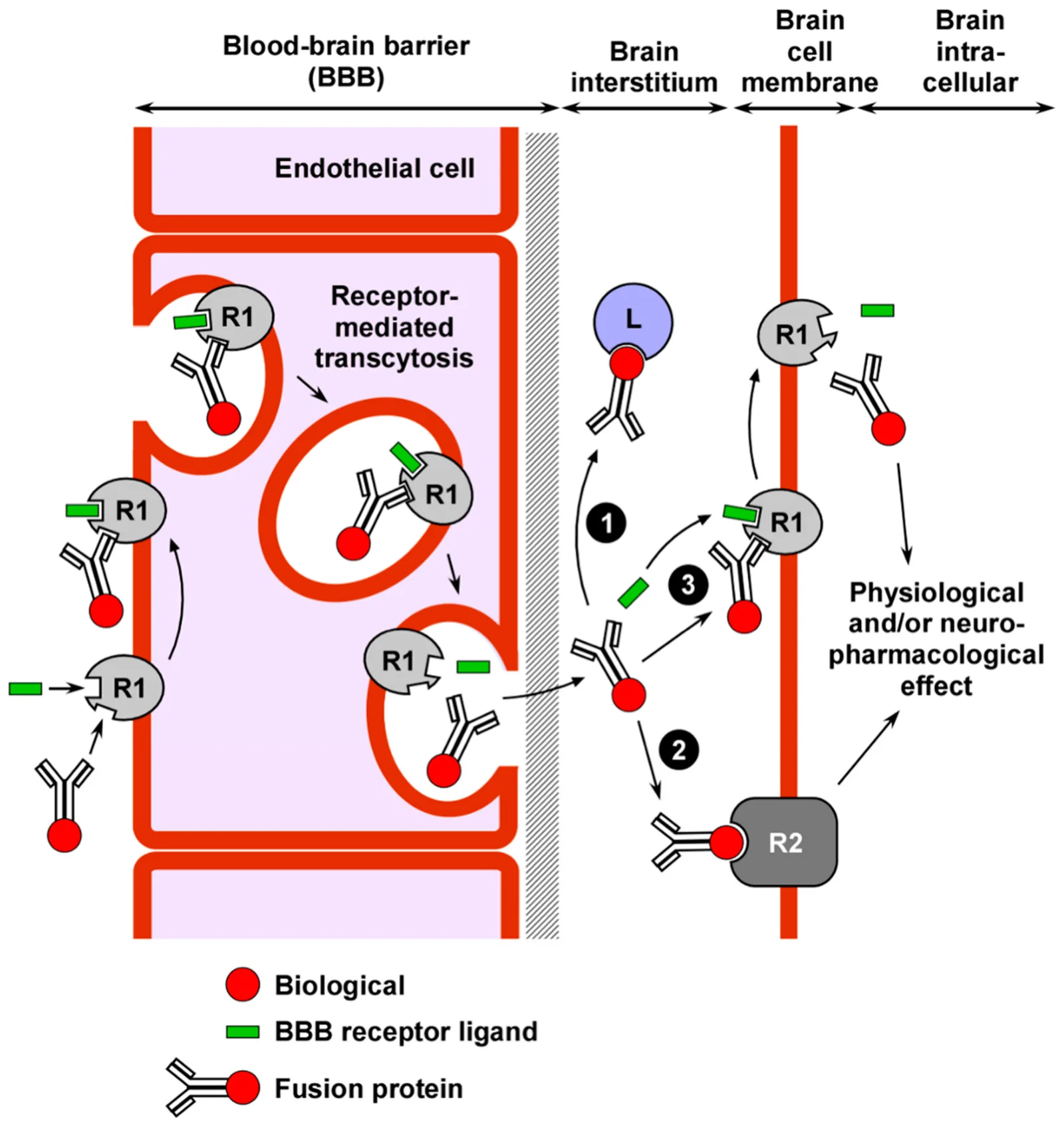
Receptor-mediated transcytosis (RMT) of antibody-based biologics across the BBB. Brain-penetrating MAbs or binding fragments of Ab recognize exofacial epitopes on endogenous BBB receptors (R1), such as the insulin receptor, transferrin receptor, or IGFR. Targeting is without interfering with the binding of the natural endogenous ligand (green rectangle) and/or the normal physiological function of the BBB by the RMT pathway. Many candidate CNS therapeutics, including enzymes, MAbs, decoy receptors, and/or neurotrophic factors (red circle), do not cross the BBB and therefore largely remain within the systemic circulation after IV administration. To facilitate CNS delivery, these therapeutic molecules can be re-engineered as fusion proteins with a brain-penetrating MAb or a MAb binding fragment, to piggyback on these BBB receptor transport systems. Following transcytosis, the mechanism of action (MoA) depends on the therapeutic domain of the fusion construct and may involve: (arrow 1) engagement of a soluble ligand (L, blue circle) within the brain interstitial compartment (as in the case of bispecific MAbs or decoy receptors); (arrow 2) binding to a brain cell membrane receptor (R2), as for neurotrophic factors; or (arrow 3) being endocytosed via the same targeted R1 receptor in brain cells, as is the case for lysosomal enzymes or antisense oligonucleotides. These processes ultimately enable the intended physiological and/or neuropharmacological response.

The brain microvasculature constitutes the blood-brain barrier (BBB) in vivo, providing a protective interface that limits central nervous system (CNS) exposure to circulating peripheral neurotransmitters, cytokines, and microorganisms, and is permeable only to small lipophilic molecules with a molecular weight below 400 Da [1,2,3,4,5,6,7]. Lipophobic nutrients of low molecular weight are transported through the BBB via receptors that form the carrier-mediated transport (CMT) family, also known as facilitated transporter systems. For example, the BBB glucose transporter type 1 (GLUT1) mediates the brain uptake of glucose, and large amino acids are transported via the BBB large amino acid transporter (LAT1) [8,9]. Although the BBB is generally impermeable to proteins, selected peripheral hormones and carrier proteins, including insulin, transferrin (Tf), insulin-like growth factor 1 (IGF-1), and leptin, can be transported across the BBB by specific receptors that undergo receptor-mediated transcytosis (RMT) through the brain microvasculature [10,11,12,13]. It was hypothesized in the mid-1980s that peptidomimetic drugs could be transported into the brain via the BBB receptors, such as the RMT systems used by insulin and Tf [10,14]. Monoclonal antibodies (MAb) directed against the extracellular domain of the BBB human insulin receptor, or the rodent BBB transferrin receptor, were produced [15,16,17]. These MAbs interact with BBB receptors without disrupting the binding of their natural endogenous ligands, thereby triggering RMT across the BBB (Figure 1) [15,16,17,18,19]. The first demonstration of pharmacological efficacy of brain-penetrating MAb conjugates was reported in the early 1990s with nerve growth factor and vasopressin intestinal peptide conjugated to the OX26 anti-rat transferrin MAb [20,21]. Subsequent studies reported additional chemical conjugates of MAb targeting BBB Tf and insulin receptors, with therapeutic effects in both rodent models and non-human primates [22,23,24,25]. Advances in genetic engineering then enabled the development of molecular Trojan horses and/or shuttle systems that generate fusion proteins comprising a transport domain and a therapeutic domain [26,27,28,29,30,31]. The transport domain is a MAb or MAb fragment that targets a receptor at the BBB to initiate RMT across the BBB, and the therapeutic domain mediates the pharmacological effect in the brain. MAb-based brain-penetrating fusion proteins have been developed and evaluated in vivo across experimental models of several neurological disorders, including lysosomal storage disorders (LSDs), stroke, Parkinson’s disease (PD), and Alzheimer’s disease (AD) (Table 1). Translation of this technology to the clinic is also underway, with clinical studies of brain-penetrating fusion proteins for LSDs and AD either completed or currently ongoing (Table 2) [32,33,34,35,36,37]. Pabinafusp alfa, a fusion protein of iduronate-2-sulfatase (IDS) and a MAb targeting BBB TfR, was the first brain-penetrating biotherapeutic approved by a regulatory agency for the treatment of Hunter’s MPSII syndrome [38]. Tividenofusp alfa was the first BBB-TfR-binding biological approved by the FDA for Hunter’s syndrome [39]. Both the biotechnology industry and academic laboratories are actively advancing the development of biologicals for brain by targeting BBB receptors. The purpose of this article is to provide an overview of the biology of BBB receptors targeted for brain delivery of MAb-based biologicals and to summarize recent progress in the field of brain-penetrating biotherapeutics.

## 2. Brain Delivery of Antibody-Based Biologicals Through Blood-Brain Barrier Receptors

### 2.1. Human Insulin Receptor (HIR)

The human insulin receptor (HIR) expressed on brain microvascular endothelial cells (BMECs) plays an important role in mediating the delivery of circulating insulin into the central nervous system (CNS). Thus, the BBB insulin receptor gained attention in the mid-1980s as a potential port of entry for biotherapeutics to the brain via receptor-mediated transcytosis [10,14]. Structurally, HIR is a disulfide-linked homodimer (αβ)_2_ and genetically is a member of the class II of receptor tyrosine kinases. Due to alternative splicing, HIR exists as two isoforms, the short form HIR-A (lacking exon 11) and long form HIR-B (containing exon 11), which differ in their C-terminal end [68]. Transport of insulin across the BBB is blood-to-brain unidirectional [69,70]. Insulin can be internalized by the signaling-related HIR present on BMECs, a process termed receptor-mediated endocytosis (RME), and transport across the BBB occurs by RMT. RME internalizes a vesicle that can return to the apical (luminal) surface; RMT incorporates the additional process of exocytosis at the basolateral (abluminal) side and is a mechanism of transport across the BBB [71].

HIR is broadly distributed in the human body, with high expression in insulin-responsive tissues such as liver, skeletal muscle, and adipose tissue [72,73]. At the BBB, HIR is localized predominantly to brain capillary endothelial cells that form the microvascular network [10]. HIR is highly enriched on the apical surface facing the circulation, where it binds circulating insulin [10,74]. A subset of HIR appears on the basolateral surface as well [10,75,76,77]. Regional variation of HIR is observed. The density of endothelial HIR varies across brain regions, with relatively higher expression observed in the hypothalamus, olfactory bulb, hippocampus, and cerebellum—regions associated with energy homeostasis, feeding behavior, and cognition [71,78,79,80]. For example, the olfactory bulb displays the highest transport rate, ~7-fold greater than the whole brain transport rate [81]. The two HIR isoforms, HIR-A and HIR-B, exhibit different ligand binding affinities and patterns of tissue distribution [82]. HIR-A, the short form, has a higher affinity for insulin (*K_D_* ≈ 0.1~0.5 nM) and is predominantly expressed in fetal tissues and the CNS. In contrast, HIR-B, characterized by lower affinity (*K_D_* ≈ 1~2 nM), predominates in metabolic tissues and regulates systemic glucose uptake [83,84]. BBB endothelial cells mainly express HIR-A, consistent with its high sensitivity to circulating insulin [10,85,86]. Ligand-receptor binding initiates HIR autophosphorylation and downstream signaling through canonical pathways, notably the PI3K-Akt and MAPK/ERK cascades [87].

#### 2.1.1. Brain-Penetrating MAbs Directed to the HIR

Murine MAbs directed against the human insulin receptor, designated 83-7 and 83-14, were produced and extensively validated in a mouse-human chimeric configuration (Table 1) [17,37]. The anti-HIR MAbs were species-specific and cross-reacted with Old World primates, such as the Rhesus monkey [10,19]. A humanized form of the 83-14 HIRMAb was also reported and validated in vivo in a Rhesus monkey [27]. HIRMAb binds their target receptors at the BBB with high affinity, exhibiting dissociation constants (*K_D_*) in the low-nanomolar range, comparable to that of the endogenous ligand insulin, while not altering physiological insulin transport across the BBB [10,11,12,13,14,15,16,17,18,19]. The brain uptake of HIRMAb, alone or as fusion proteins or conjugates, was high at 1–3% injected dose (ID)/brain [37,40], a value that is comparable to the brain uptake of diazepam, a small, lipid-soluble molecule that penetrates the brain in a single pass [88]. HIRMAb and HIRMAb-fusion proteins also exhibit relatively large brain volume of distribution (VD), ranging from ~300 to 800 μL/g [37,40], as compared to the brain VD of non-BBB-penetrating IgGs, which is equivalent to the blood volume in the brain, i.e., 14–20 μL/g [41,89]. The BBB penetration of HIRMAb and HIRMAb-fusion proteins was also quantified by the capillary depletion technique, demonstrating transport across the BBB and not retention in the brain endothelial cells [37,40,41,89].

#### 2.1.2. Biodistribution of a Brain-Penetrating HIRMAb-Iduronidase (IDUA)

**Figure 2:**
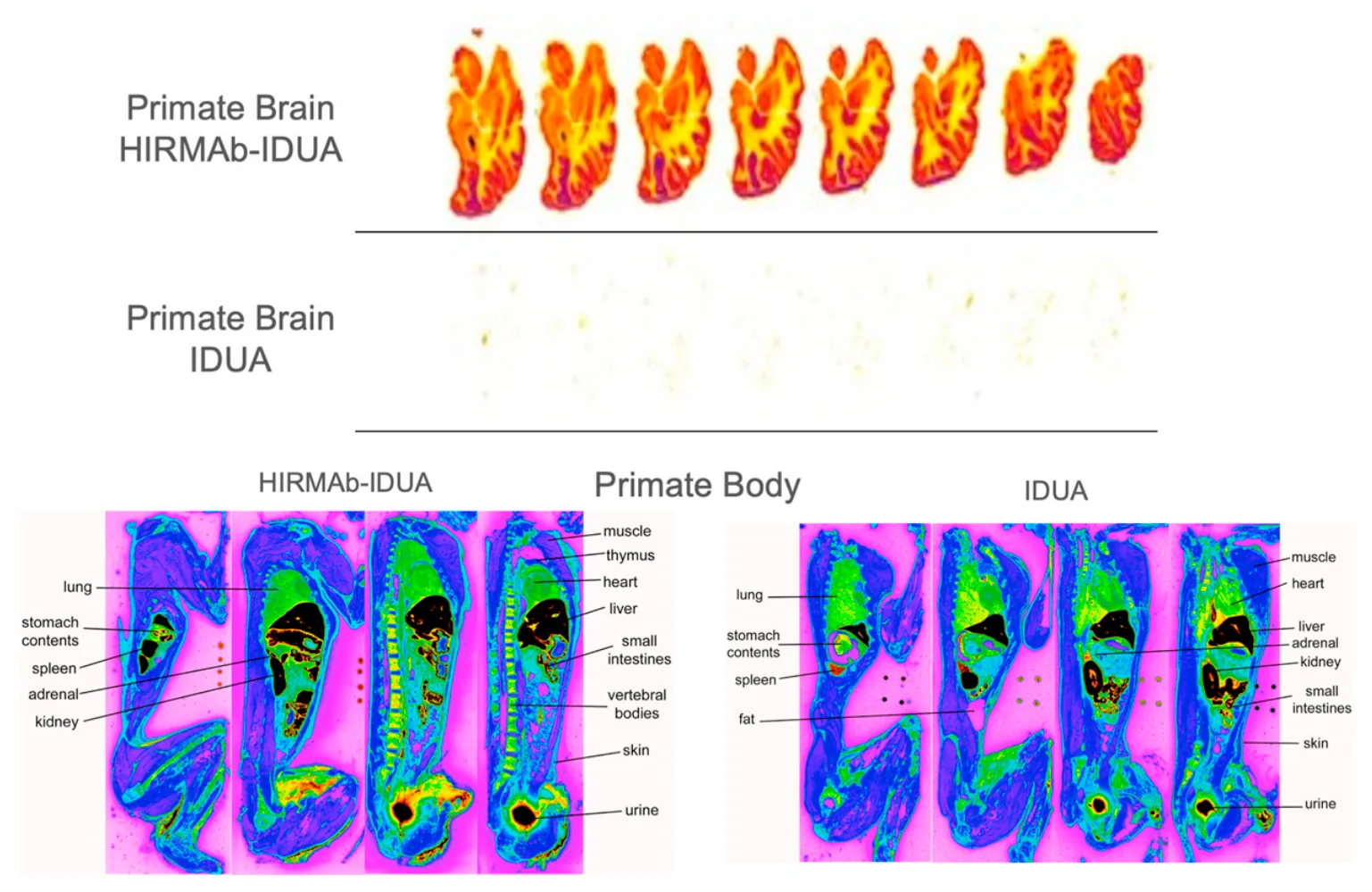
Transport of HIRMAb-IDUA (valanafusp alpha, AGT-181) across the BBB via the insulin receptor and to peripheral organs in a Rhesus monkey. The upper two panels display 8 parallel sagittal sections of a rhesus monkey cerebral hemisphere obtained 2 h after IV administration of [^125^I]-HIRMAb-IDUA fusion protein or recombinant [^125^I]-IDUA, as indicated. The cerebellum can be identified in the medial sections of the brain (**top left**). The BBB-penetrating HIRMAb-IDUA shows widespread distribution throughout the brain, as indicated by the yellow–orange signal, whereas recombinant IDUA does not cross the BBB and shows only background activity in the brain. The distribution of IDUA (**bottom right**) and HIRMAb-IDUA (**bottom left**) in peripheral organs is comparable, as both proteins are taken up through the M6P receptor in peripheral organs. There is, however, an increase in the uptake of HIRMAb-IDUA in bone marrow and vertebral bodies compared with recombinant IDUA (from ref. [37]).

HIRMAb-IDUA is perhaps the most well-characterized brain-penetrating IgG-fusion protein targeting the BBB insulin receptor. It was the first molecule in its class to complete a phase I/II clinical trial in pediatric Hurler’s syndrome MPSI (Section 2.1.3). HIRMAb-IDUA (valanafusp alpha, AGT-181) is a fusion protein engineered for the potential treatment of Hurler’s syndrome MPSI [90]. In HIRMAb-IDUA, the chimeric HIRMAb is fused to the human IDUA at the C-terminus of the heavy chain of the transport MAb targeting the BBB insulin receptor [37,91,92]. The HIRMAb-IDUA maintained high affinity for the HIR and a specific enzyme activity comparable to the mature recombinant IDUA [92]. The HIRMAb-IDUA markedly reduced the accumulation of glycosaminoglycans (GAG) in the lysosomal compartment of Hurler’s fibroblasts [92]. In vivo biodistribution of HIRMAb-IDUA was investigated in the Rhesus monkey and compared with that of IDUA alone (Figure 2). A global distribution throughout the brain was seen with the HIRMAb-IDUA, whereas IDUA alone showed background activity, as it does not cross the BBB (Figure 2). The brain VD of HIRMAb-IDUA was high at >700 mL/g brain [89]. On the contrary, the brain VD of recombinant IDUA’s was low and comparable to the blood volume in the brain [89]. The brain penetration of HIRMAb-IDUA was also demonstrated by the capillary depletion method [89]. The distribution of the HIRMAb-IDUA in peripheral organs was like the distribution of the recombinant IDUA alone, as both are taken up in the peripheral organs through the mannose-6-phosphate (M6P) receptor (Figure 2) [89]. There was, however, an increase in the uptake of HIRMAb-IDUA in vertebral bodies and bone marrow as compared with recombinant IDUA (Figure 2) [89]. Based on the reported brain uptake of HIRMAb-IDUA (1.2% ID/brain), administration of the fusion protein at 1 mg/kg BW has been estimated to provide sufficient brain exposure to restore IDUA levels toward the normal range in individuals with Hurler syndrome [89]. At this dose, the predicted brain concentration is ~3 ng/mg brain protein, corresponding to an IDUA enzymatic activity of ~1.1 units/mg brain protein [89], which is comparable to physiological IDUA levels reported in human brain [93]. This hypothesis is supported by studies in mouse models of MPSI showing efficacy with fusion proteins targeting the mouse or the human TfR and fused to IDUA [54,94].

#### 2.1.3. Phase I/II Clinical Trial with Valanafusp Alpha (HIRMAb-IDUA, AGT-181) in Pediatric Hurler’s Syndrome MPSI

**Figure 3:**
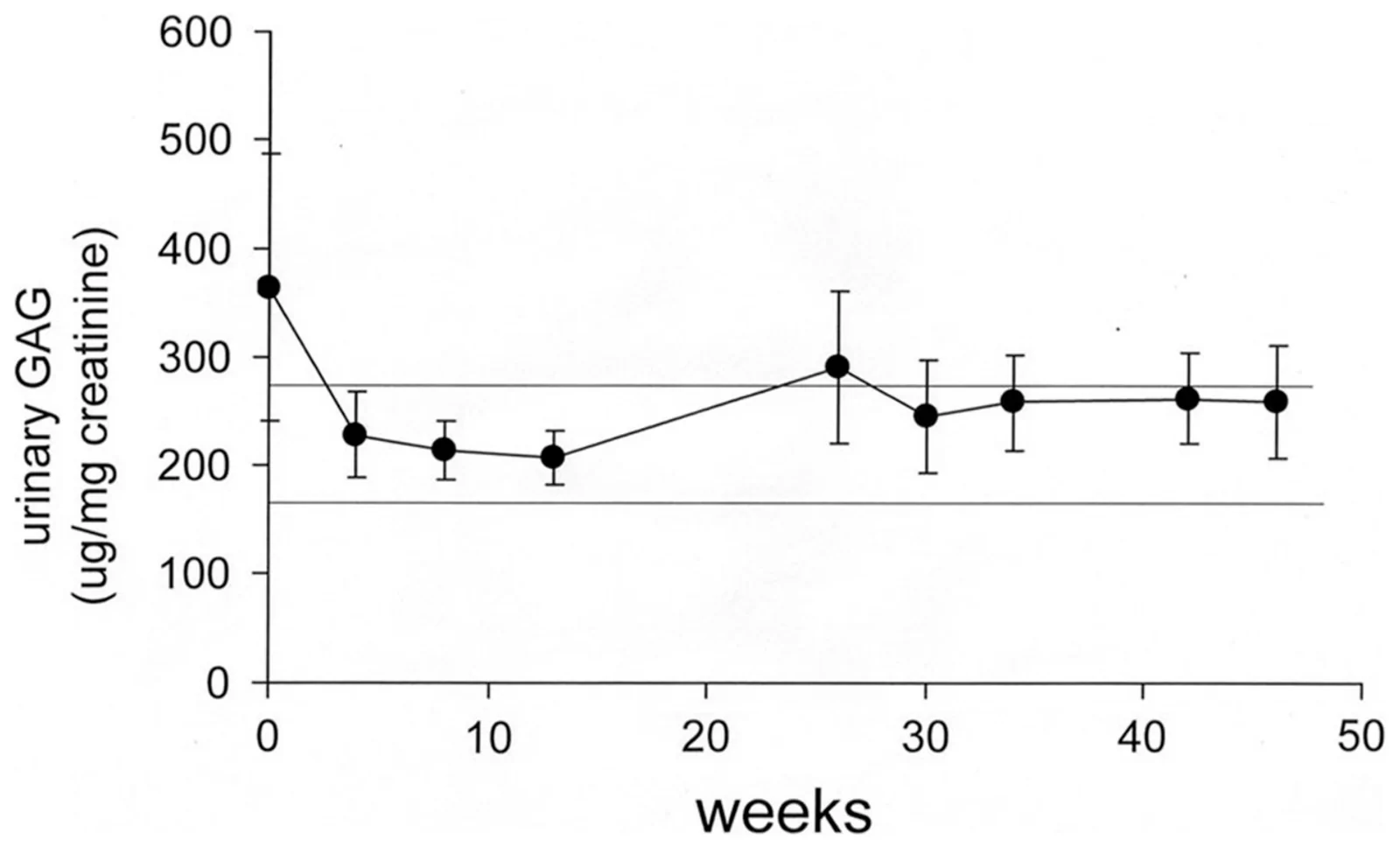
Stabilization of urinary GAG levels in pediatric subjects with MPSI during 52 weeks of valanafusp alpha (HIRMAb-IDUA) treatment. Urinary GAG concentrations are presented as mean ± SEM at the indicated treatment weeks. For comparison, the horizontal reference lines represent the mean ± SEM reported for 12 children with MPSI following 12 months of laronidase therapy; urinary GAG concentrations in this cohort ranged from 177 to 269 ug GAG/mg creatinine [95] (from ref. [32]).

Valanafusp alpha was the first brain-penetrating molecule of its class to complete a clinical trial in Hurler’s syndrome MPSI (Table 2) [32]. Patients, previously treated with laronidase, were dosed with valanafusp alpha at 1–6 mg/kg weekly IV for up to 12 months. The urinary GAGs were stabilized throughout the 52 weeks (Figure 3) [32]. A dramatic reduction in liver and spleen volumetrics was also reported (Table 3) [32]. This observation may reflect the dual targeting of valanafusp alpha in the peripheral organs via both insulin- and M6P receptor-mediated pathways [32,89]. After 26 weeks of valanafusp alpha therapy, a significant increase in shoulder flexion and extension was also observed (Table 3). These musculoskeletal benefits may be related to the preferential targeting of HIRMAb-IDUA to vertebral bodies and joints, as previously demonstrated in primates (Figure 2) [89]. In addition, patients with either severe or attenuated MPSI who received valanafusp alpha showed mean improvements across all evaluated cognitive domains relative to the natural history outcomes reported for untreated MPSI patients [32].

Using the same HIRMAb delivery system, a brain-penetrating form of PPT1 was engineered for the potential treatment of Batten Type 1 disease or CLN1 disease by fusion of PPT1 [46,96]. A child with CLN1 disease was dosed with HIRMAb-PPT1 under compassionate use in Germany for 26 months at 2.6 mg/kg BW weekly [97]. Treatment with the fusion protein resulted in improved quality of life with better epilepsy control and no adverse effects [97].

#### 2.1.4. Verenafusp Alfa (HIR-Fab-IDS, GNR-055)

Another construct targeting the BBB HIR was reported as a potential therapeutic approach for MPSII [42]. Contrary to the HIRMAb design of valanafusp alpha, this molecule consists of a monovalent Fab fragment engineered to target the BBB HIR and fused to human IDS through a short LSS linker, i.e., HIR-Fab-IDS (GNR-055) [42]. The HIR-Fab-IDS fusion protein retains high-affinity binding to HIR (*K_D_* = 0.136 nM) while preserving IDS activity comparable to that of recombinant IDS [42]. The brain uptake in the cynomolgus monkey was comparable to that of HIRMAb at 1% ID/brain. The brain and peripheral organs distribution of HIR-Fab-IDS was similar to that of HIRMAb-IDUA shown in Figure 2 [42]. Clinical trials with HIR-Fab-IDS (GNR-055, verenafusp alfa) are in progress, and data in healthy volunteers from an open-label multicohort phase I study were recently reported (Table 2) [98].

### 2.2. Human Transferrin Receptor (TfR)

Targeting the BBB TfR has gained the attention of both academia and the biotech industry over the last few decades, representing the most well-characterized BBB RMT system. Today, most of the programs developing biologics for the CNS are focused on targeting the BBB TfR (Table 2). The endothelial transferrin receptor 1 (TfR1), also known as CD71, on the BBB plays a role in the physiologic regulation of iron transport into the brain, since iron is essential for brain development and neuronal function. The major systemic iron carrier, Tf, binds two ferric ions (Fe^3+^) and delivers them to cells through RME. TfR1 is a type II transmembrane glycoprotein composed of a short N-terminal cytoplasmic tail, a single transmembrane helix, and a large extracellular ectodomain that forms the canonical transferrin-binding site. The cytosolic tail contains a conserved tyrosine-based internalization motif (YTRF) that recruits the AP-2/clathrin machinery and mediates rapid RME. The ectodomain, comprising the protease-like, apical, and helical domains, binds diferric-loaded transferrin (holotransferrin or holoTf) molecules per TfR1 homodimer to initiate RME [99,100,101]. The Tf-TfR1 complex is internalized into clathrin-coated vesicles and trafficked to Rab5-positive early endosomes, where lumen acidification (pH ~5.5) triggers iron release from transferrin. The released ferric ion (Fe^3+^) is reduced to ferrous iron (Fe^2+^) by ferrireductases and transported into the cytoplasm via a metal transporter, while iron-free transferrin (apotransferrin or apoTf) remains bound to TfR1. The apoTf-TfR1 complex then recycles back to the plasma membrane through recycling endosomes, where neutral pH promotes dissociation, allowing TfR1 to re-enter another round of iron uptake. This continuous cycle of clathrin-mediated endocytosis, endosomal sorting, and rapid recycling underlies the efficiency of cellular iron acquisition [99,100,101]. Since the BBB controls iron entry into the CNS, the TfR expressed on BMECs is therefore a critical node coupling peripheral iron status to brain iron homeostasis and represents the principal physiologic pathway for Tf-bound iron to cross the BBB.

TfR1, encoded by the TFRC gene, is highly expressed on brain capillary endothelial cells in many mammalian species, including humans. TfR is detectable on both the luminal (blood-facing) and abluminal (brain-facing) membranes of BMECs, enabling apicobasal capture and release of transferrin during transcytosis [11,15,75,102,103,104]. Expression levels are developmentally regulated, and neonatal/juvenile brain microvessels have higher TfR density than adult brain in many species, consistent with increased iron demands during development [105,106,107]. The interaction of TfR with Tf is high affinity and modulated strongly by iron loading (apoTf vs. monoTf vs. holoTf) and by pH [108,109]. Diferric transferrin (holoTf) binds TfR with substantially higher affinity than apoTf, and affinities reported in the literature span a range depending on experimental method, labeling, and species—commonly cited *K_D_* values for holoTf binding to TfR are in the low- to sub-nanomolar up to ~10^−7^ M range depending on conditions; for example, intact cell assays [110], surface plasmon resonance (SPR) [111], or iodinated ligand binding [112] yield different numeric values. The receptor–ligand complex undergoes endocytosis; within the acidified endosome (pH ≈ 5.5–6.0), iron dissociates from transferrin, after which apoTf remains bound until receptor recycling at neutral pH releases apoTf into the extracellular space. The pH-dependent conformational changes that potentiate iron release and alter receptor affinity are well documented in structural and biochemical studies.

#### 2.2.1. Brain-Penetrating MAbs Directed to the TfR

The TfR at the BBB also gained attention in the mid-1980s as a potential port of entry to the brain through RMT [11,14]. MAbs directed against the BBB TfR1 initially produced were species-specific, i.e., OX26 MAb targeting the rat TfR, as well as 8D3 and R17 MAbs for the mouse TfR [15,16,18]. These MAbs bind BBB TfR with high affinity in the low nM range, like the affinity of transferrin for TfR1 [15,16,18]. These MAbs targeting BBB receptors allowed demonstration of transport of biologics across the BBB, producing high levels of brain uptake and brain VD [20,21,22,23,25,37,40]. Further engineering of a chimeric anti-TfR MAb [30] enabled production of various fusion proteins (Table 1). Based on tissue culture experiments performed on TfRMAb-avidin fusion proteins, which may induce clustering and degradation of the TfR [113,114,115], low-affinity monovalent TfR-MAbs were produced to improve brain uptake [35,36,57,60,113]. However, no evidence of downregulation of BBB TfR was reported in vivo with a high-affinity TfRMAb fusion protein in a chronic study [116]. Moreover, no evidence of BBB TfR downregulation was reported either in a chronic study performed with the high-affinity human bivalent TfRMAb-IDS fusion protein, pabinafusp alfa, in the cynomolgus monkey with doses up to 30 mg/kg/week for 26 weeks [117].

Kinetics modeling of transport across the BBB with TfR produced different results. A publication reported an optimal BBB TfR receptor-binding *K_D_* of 0.5–5 nM for TfRMAb, resulting in a therapeutic dose of 3 mg/kg BW [118]. A lower therapeutic dose may be preferred to reduce potential adverse effects [49,119]. On the contrary, other modeling predicted better brain uptake with monovalent low-affinity MAb targeting the BBB TfR [120]. This created two tendencies in the development of brain-penetrating biotherapeutics targeting the BBB TfR, i.e., (a) high-affinity full or monovalent TfR MAbs, like pabinafusp alfa (Section 2.2.2) or lepunafusp alfa (Section 2.2.3), or (b) low-affinity monovalent TfR MAbs or engineered binding sites for TfR, like tividenofusp (Section 2.2.4), and trontinemab (Section 2.2.5) (Table 1 and Table 2). Recent publications also aimed to fine-tune the affinity of MAb targeting BBB receptors for brain delivery of biologics [121,122,123,124].

#### 2.2.2. Pabinafusp Alfa (hTfRMAb-IDS, JR-141)

Pabinafusp alfa is a brain-penetrating IDS fusion protein engineered to bind the BBB TfR with high affinity (*K_D_* = 1.22 nM) in a dimeric configuration, and it was the first of its class to be approved for Hunter’s MPSII syndrome in Japan [38,125] (Table 2). In a hTfR knock-in mouse model of Hunter’s syndrome, intravenous administration of pabinafusp alfa at 1 mg/kg BW was effective in reducing GAGs in brain and peripheral organs [125]. Phase I/II clinical trials in patients with pabinafusp alfa in Hunter MPSII subjects reported reduction in the levels of heparan sulfate (HS) concentrations in plasma, urine, and CSF, with minor adverse reactions [126,127]. In a phase II/III clinical trial in MPSII, weekly intravenous treatment with pabinafusp alfa at 2 mg/kg BW for 52 weeks resulted in a significant decrease in the CSF HS levels, which was used as the primary efficacy endpoint [34]. Further evaluation of pabinafusp alfa in patients with Hunter’s syndrome MPSII is ongoing in an extended phase II/III clinical study (NCT04573023) (Table 2).

Using the same brain high-affinity penetrating shuttle system, a fusion protein comprising hTfRMAb and NAGLU (JR-446) was produced, and a phase I/II clinical trial in Sanfilippo MPSIIIB is in progress [59] (Table 2).

#### 2.2.3. Lepunafusp Alfa (hTfR-Fab-IDUA, JR-171)

Lepunafusp alfa was engineered for the treatment of Hurler’s syndrome MPSI using the same high-affinity MAb directed to the BBB TfR as in pabinafusp alfa, but in a monomeric configuration [33,94] (Table 1 and Table 2). Even though it has a single binding domain for the BBB TfR, lepunafusp alfa binds the human TfR with high affinity, i.e., *K_D_* = 1.88 nM [33,94]. In an animal model of MPSI, lepunafusp alfa significantly reduced the levels of dermatan sulfate (DS) and heparan sulfate (HS) in brain and peripheral organs [94]. Another mouse-specific TfRMAb-IDUA fusion protein produced similar effects [54]. In peripheral organs, lepunafusp alfa was as effective as recombinant IDUA, but also improved spatial learning ability [94]. Lepunafusp alfa was well tolerated and safe in a phase I/II trial in MPSI subjects, reducing serum and urinary GAG, HS in CSF, and was associated with positive neurocognitive and behavioral changes [33] (Table 2).

Using the same brain high-affinity monomeric shuttle system, a fusion protein comprising hTfRMAb-Fab and SGSH (postnafusp alfa, JR-441) was produced, and a phase I/II clinical trial in Sanfilippo MPSIIIA is in progress [56] (Table 2).

#### 2.2.4. Tividenofusp Alpha, Enzyme Transport Vehicle, ETV:IDS (DNL310)

Another brain shuttle system was engineered to target the BBB TfR and designated Enzyme Transport Vehicle (ETV). The transport domain in EVT is human IgG1 hinge-CH2-CH3 Fc fragment-bisdisulfide with a human IgG1 hinge-CH2-CH3 Fc fragment-bisdisulfide engineered for binding to the human TfR1 with very low affinity (i.e., *K_D_* = 150–300 nM) [36,128]. EVT was used to construct a fusion protein with IDS, EVT:IDS, or tividenofusp alpha, for MPSII [36]. The human pro-IDS is fused to EVT through a short linker to its N-terminus [36]. A marked reduction in brain and peripheral organs GAGs was reported [36]. Phase II/III clinical trials are in progress with tividenofusp alfa in Hunter’s syndrome MPSII subjects (Table 2). Tividenofusp alfa was recently approved by the FDA, establishing it as the first BBB-TfR-binding biological for Hunter’s syndrome approved in the USA [39].

Using the same EVT delivery system, fusion proteins were engineered for Sanfilippo A MPSIIIA (EVT:SGSH, DNL126) and Pompe disease (ETV:GAA, DNL952), respectively [57,60]. An open-label phase I/II clinical trial of EVT-SGSH is ongoing in patients with Sanfilippo MPS IIIA (Table 2). A clinical study to evaluate the safety, pharmacokinetics, and pharmacodynamics is planned with EVT:GAA in adults with late-onset Pompe disease (Table 2).

#### 2.2.5. Trontinemab (TfR-scFab-Aβ-MAb)

Trontinemab is the first brain-penetrating biological targeting the BBB TfR, completing a phase I/II clinical trial for the immunotherapy of AD [35] (Table 2). Trontinemab is engineered as a recombinant (2 + 1) bispecific humanized MAb anti-Aβ (gantenerumab) and fused to an anti-TfR Fab fragment, which binds the TfR with low affinity (*K_D_* = 131 nM) in monomeric configuration [35]. Preliminary data from a phase I/II clinical study in AD showed a marked reduction in the levels of amyloid in the brain by amyloid positron emission tomography. AD biomarkers, amyloid and total and phosphorylated Tau, were also reduced in CSF. Trontinemab showed a safety profile, with ARIA occurring in <5% of patients, and no new cases have been reported [129].

#### 2.2.6. Other Brain-Penetrating Fusions Targeting the BBB TfR

More recently, a BBB TfR-targeting antibody transport vehicle (ATV) has been developed to facilitate antibody-based immunotherapy for AD [130]. This anti-Aβ MAb encompasses the same low-affinity TfR-binding domain as the one described above for ETVs, and asymmetrical Fc mutations (ATV^cisLALA^) engineered to mitigate hematological adverse effects associated with TfR engagement [130]. This molecular design presented virtually complete elimination of ARIA lesions and vascular inflammation [130].

Several additional brain-penetrating constructs or shuttle systems and fusion proteins targeting the BBB TfR have been produced and validated preclinically in animal models of CNS disorders (Table 1). A shark-derived anti-TfR1 single-domain antibody targeting TfR1 fused to a TrkB agonist antibody provided full neuroprotection in the 6-hydroxydopamine mouse model of PD [131,132]. The following fusion proteins engineered with a high affinity chimeric anti mouse TfR based on the 8D3 MAb [30] were constructed and validated: (a) mTfRMAb-IDUA in a mouse model of MPSI [54]; (b) mTfRMAb-SGSH in a mouse model of MPSIIIA [58]; (c) mTfRMAb-Aβ bi-specific antibody in a mouse model of Alzheimer’s disease [61]; and (d) mTfRMAb-TNFR, mTfRMAb-EPO and/or mTfRMAb-GDNF in mouse models of Parkinson’s, Alzheimer’s, and/or stroke [37,39,62,63,64,133]. The amino acid sequence of the 8D3 anti-mouse TfR MAb was employed to engineer a tetravalent MAb based on the 8D3 sequence with a recombinant variant of the full IgG mAb158 (RmAb158) [134]. In this construct, the 8D3 MAb was incorporated as an scFv fused to the C-terminus of the RmAb158 light chain [66,135]. The resulting RmAb158-scFv8D3 demonstrated efficacy in a mouse model of AD, as evidenced by both imaging studies and reduction of soluble Aβ protofibrils [135]. In addition, a monovalent scFv8D3 fused to Aβ-affibodies was also reported to produce global distribution throughout the brain [136]. A TfR-tetravalent bispecific tandem-Aβ IgG was also shown to be effective in an AD animal model, without serious adverse effects on hematological parameters and organ histopathology following chronic treatment [137]. The rat anti-mouse TfR 8D3 MAb in scFv format was fused to somatostatin (SST), a peptide known to increase the activity of neprilysin (NEP). This zinc-dependent metalloprotease cleaves monomeric and pathological oligomeric Aβ40 [138,139]. The 8D3-scFv-SST fusion protein increased the brain endogenous levels of NEP, inducing degradation of membrane-bound Aβ42 in an animal model of AD [140]. A brain-penetrating NEP fusion protein was also engineered with a high-affinity mouse anti-rat OX26 TfR MAb in a monomeric format fused to the constant region of IgG1, and with the NEP fused to the C-terminus of the transport construct [140]. The TfR-sFab-Fc-NEP construct retained enzymatic activity and exhibited high-affinity binding to the rat TfR, comparable to the high affinity of the dimeric OX26 [140]. This fusion protein reached a brain concentration of approximately 110 ng/mL at 24 h after the IV injection of 10 mg/kg BW, resulting in a significant reduction in Aβ40 [140].

### 2.3. Insulin-like Growth Factor-1 Receptor (IGF1R)

Insulin-like growth factor-1 receptor (IGF1R) has also been investigated as a potential BBB transport of biotherapeutics [141,142]. IGF1R belongs to the receptor tyrosine kinase (RTK) family and primarily mediates the biological actions of insulin-like growth factor 1 (IGF1). IGF1R binds IGF1 with high affinity, whereas insulin-like growth factor 2 (IGF2) and insulin bind with low affinity [143]. Ligand binding activates the RTK, leading to receptor autophosphorylation, tyrosine phosphorylation of multiple substrates, and subsequent activation of two principal signaling cascades: the phosphoinositide 3-kinase (PI3K)-Akt/PKB pathway and the Shc-Ras-MAPK pathway [144]. Through these signaling networks, IGF1R contributes to normal growth and development as well as plays an important role in maintaining neuroplasticity [145,146]. Structurally, IGF1R is a highly glycosylated transmembrane receptor organized as a disulphide-linked (αβ)2 heterotetramer. Its α and β subunits are stabilized by tertiary interactions together with inter- and intra-chain disulphide bonds [147,148].

IGF1R is broadly distributed throughout the body, although its expression in the brain is reported to be modestly higher than that in peripheral tissues [74,149]. At the BBB, IGF1R is accessible from the blood-facing, or luminal, surface of brain endothelial cells [150]. It also demonstrates a degree of regional distribution, with relatively high levels detected in the developing cerebellum, midbrain, olfactory bulb, and in the ventral floor plate of hindbrain [151]. High IGF1R expression has been identified in brain endothelial cells (BECs) and isolated brain microvessels (BMVs) from mice and humans [74], as well as in neuronal cells within specific brain regions [152]. Functionally, IGF1R participates in the delivery of circulating ligands, including IGF1 and insulin, to brain regions such as the hypothalamus and hippocampus [153,154,155]. The transport of circulatory IGF1 across the BBB in vivo is modulated by local neuronal activity [156]. The binding and endocytosis of IGF2 is greater than that of IGF1 in isolated human brain capillaries [13]. In addition, brain and cerebrospinal fluid (CSF) levels of IGF2 are considerably higher than those of IGF1 [157]. If the IGF1R on the BBB acts as a transport system, then it would be expected that the receptor is more active for IGF2 than for IGF1 [13]. However, according to the purified Chinese hamster ovary cell CHO-K1-derived leucine-zippered stabilized human IGF1R ectodomain, the reported affinity (IC50) was 0.39 nM. (0.33–0.47 nM) for IGF1, compared with 1.24 nM (1.05–1.46 nM) for IGF2 [158]. In addition, human IGF1 binds IGF1R with substantially greater affinity (*K_D_* = 0.2984 nM) than human insulin (*K_D_* = 293 nM) [159].

Lama single-domain antibodies (sdAbs) directed to the IGF1R were produced with high binding affinity for human IGF1R (*K_D_* ~0.3 to 1.3 nM) and a brain uptake of 12 nM 24 h after the IV administration of 15 mg/kg BW [141,142]. In vivo efficacy was demonstrated with neurotensin fused to the lama transport IGF1R5-hFc vector, inducing a dose-dependent decrease in core temperature. In addition, galanin chemically conjugated to IGF1R5-mFc reversed hyperalgesia [141,142]. In addition, the IGF1R-based vector also enabled effective transport of IDS in the rat brain [67].

## 3. BBB Carrier-Mediated Transport (CMT) of Nutrients

The other class of transporters at the BBB are the facilitated transporters or carrier-mediated transporters (CMTs). These CMTs allow for the passage across the BBB of small hydrophobic nutrient molecules like glucose and amino acids, but do not induce RMT. Examples of the BBB CMTs are GLUT1 (encoded by SLC2A1) and the BBB LAT1 (encoded by SLC7A5) [8,9]. The classification and function of CMTs have been extensively reviewed [1,160]. The transport of radiopharmaceuticals and prodrugs across the BBB and through CMT has been successfully employed throughout several decades. For example, ^18^F-fluorodeoxyglucose is transported to the brain via the BBB-GLUT1 for imaging of brain tumors by PET [161]. L-DOPA, a widely used therapy for Parkinson’s disease, reaches the brain through the BBB-LAT1 [162,163]. The targeting of the BBB-GLUT1 and the heavy chain of the BBB-LAT1 (CD98hc or SLC3A2) has been proposed as alternative molecular pathways for shuttling therapeutic agents across the BBB [164,165]. More recently, an antibody transport vehicle targeting CD98hc (ATVCD98hc) has continued to be developed as a potential platform for receptor-mediated delivery of biologics to the brain [166].

## 4. Discussion

The data reviewed in the present article are consistent with the following conclusions. First, it is possible to develop antibody-based biologics for the brain by targeting BBB receptors. Second, the field of brain drug delivery of biotherapeutics via BBB receptors has evolved over the last 4 decades from antibody conjugates to the engineering of brain-penetrating MAb-fusion proteins currently in clinical trials [14,37] (Table 1 and Table 2). Third, targeting the insulin receptor, valanafusp alfa was the first brain-penetrating biological completing a phase I/II clinical trial in MSPI (Section 2.1.3). Fourth, targeting the transferrin receptor, pabinafusp alfa was the first brain-penetrating biotherapeutic receiving regulatory approval for MPSII in Japan (Section 2.2.2). Fifth, targeting the transferrin receptor, tividenofusp alfa was the first BBB-TfR-binding biological approved by the FDA for Hunter’s syndrome (Section 2.2.4). Sixth, targeting the transferrin receptor, trontinemab was the first brain-penetrating biological completing a phase I/II clinical trial for the immunotherapy of AD (Section 2.2.5). Seventh, taking into consideration pre-clinical studies and ongoing clinical trials, the development of brain-penetrating biologics is mainly focused on the BBB TfR, and to a lesser extent the BBB insulin and IGF1 receptors, respectively (Section 2.1, Section 2.2 and Section 2.3).

Multiple antibody-fusion proteins targeting the human IR have been engineered and validated in Rhesus monkeys (Table 1) [37]. Based on studies in primates, therapeutic brain levels of lysosomal storage enzymes may be achieved following the IV administration of 1–3 mg/kg BW HIRMAb-IDUA for Hurler’s MPSI [89,92], HIRMAb-IDS for Hunter’s MPSII [42], HIRMAb-ASA for MLD [43], HIRMAb-SGSH for Sanfilippo MPSIIIA [44], and HIRMAB-NAGLU for Sanfilippo MPSIIIB [45], among other LSDs (Table 1) [37,46]. Along the same line, dosing with HIRMAb-Aβ bi-specific antibody, HIRMAb-TNFR decoy receptor, HIRMAb-EPO and/or HIRMAb-GDNF may be therapeutic in other CNS disorders like Alzheimer’s, Parkinson’s and/stroke, respectively (Table 1) [37,47,48,49,50]. Chronic targeting of the insulin receptor with HIRMAb fusion proteins was safe, as GLP pharm/tox studies in primates showed no toxicity and few acute hypoglycemic episodes [37,40], also seen in the phase I/II clinical trial with valanafusp alfa in pediatric MPSI, representing an incidence of just 2.8% within the therapeutic dose [32]. Valanafusp alfa was well tolerated by Hurler’s pediatric subjects without serious adverse effects and just 10 infusion-related reactions in >570 test article infusions [32]. Despite extensive safety and efficacy in humans and nonhuman primates, further development of BBB-penetrating biologics, as well as clinical trials, was focused on targeting the BBB transferrin receptor (Table 1 and Table 2), except for HIR-Fab-IDS for Hunter’s syndrome MPSII [41,98]. Availability of hTfR knock-in mice [125] presents an advantage in the development of biologics for the brain as compared with the BBB insulin receptor, since the same construct is used in preclinical murine studies and in human clinical trials. HIR knock-in may be produced in mice; however, brain uptake in those animals will be suboptimal, as the abundance of the IR in mice is markedly downregulated compared with the TfR [167,168].

The targeting of the BBB TfR with brain-penetrating antibody-based biologics presented a good safety profile as well. A reduction in circulating reticulocytes was observed after acute dosing with a low-affinity TfRMAb, and the mutation of the Fc effector function rescued the adverse effect [169]. Conversely, chronic studies using high-affinity TfRMAb did not report changes in circulating reticulocytes, suggesting that the effect on reticulocytes appears to be transient and reversed by chronic treatments [37]. In addition, no effector function was reported with pabinafusp alfa in cynomolgus monkey chronic studies with doses up to 30 mg/kg/week for 26 weeks [117]. A clinical trial with pabinafusp alfa in MPSII patients further demonstrated transient infusion-related reactions, which were clinically manageable and did not require discontinuation of the treatment. Serious adverse events in five patients were reported, and those were unrelated to the test drug [34]. Trontinemab demonstrated a good safety profile in a phase I/II clinical trial as well, with ARIA observed in <5% of participants. These findings were most likely associated with the therapeutic Aβ MAb rather than the TfR-Fab transport domain (Section 2.2.5).

Based on current developments and clinical trials, progress in the field of drug delivery to the brain via BBB receptors has focused on the BBB transferrin receptor (Table 2). Targeting this receptor, different vectors have been developed and are being tested in the clinic. Major differences relate to the affinity for the TfR. For example, pabinafusp alfa and lepunafusp alfa bind to the BBB TfR with high affinity, in dimeric or in monomeric format, respectively [33,34]. On the contrary, trontinemab and tividenofusp alpha target the BBB TfR with low affinity [35,57]. Low-affinity shuttles showed longer plasma residence time, as opposed to the short plasma residence time described with high-affinity transport systems. The kinetics modeling suggests that the lower the affinity of a shuttle system targeting the BBB TfR, the greater the ID required to maintain a given brain AUC [118]. Indeed, the therapeutic dose of the high-affinity pabinafusp alfa is 2 mg/kg BW/week, and that of the low-affinity tividenofusp alfa is 15 mg/kg BW [127,170]. Trontinemab showed efficacy when administered at a relatively low dose, i.e., 3.6 mg/kg BW every 4 weeks [35]. Regardless of the differences in the design of shuttle systems targeting the BBB TfR, pabinafusp alfa, trontinemab and tividenofusp alfa are advancing into phase III clinical trials, with the latter having just been approved by the FDA for Hunter’s syndrome MPSII (Table 2).

## 5. Conclusions

A historical review is presented from the original hypothesis suggesting that peptidomimetic drugs may be transported into the brain through RMT systems at the BBB to the approval of the first in its class of brain-penetrating biologicals, pabinafusp alfa and tividenofusp alfa. A broad range of brain-penetrating antibody-based fusion proteins targeting various BBB receptors have been discussed. These fusion proteins were validated biochemically and in animal models of CNS disorders and continued to be evaluated in human clinical trials. From the known BBB receptors suitable for drug delivery of biologicals to the brain via RMT, the BBB TfR was mostly selected by both academia and industry for further development of the field. With the recent approval of tividenofusp alfa by the FDA for Hunter’s syndrome, and multiple ongoing clinical trials with brain-penetrating biologicals, it is anticipated that the family of antibody-based BBB-crossing molecules discussed in the present review is poised to emerge as a novel class of therapeutics for human CNS disorders in the near future.

## Figures and Tables

**Table 1:** Antibody-based fusion proteins capable of penetrating the brain by targeting BBB receptors that induce RMT across the BBB.

BBB Receptor	Indication/Therapeutic Domain	Antibody-BasedFusion Protein ^1^	Reference
Human Insulin Receptor (HIR) *	Hunter syndrome (MPS I)/Iduronidase (IDUA)	HIRMAb-IDUA (valanafusp alpha)	[32]
	Hunter syndrome (MPS II)/Iduronate-2-sulfatase (IDS)	HIRMAb-IDS	[40,41]
	Hunter syndrome (MPS II)/IDS	HIR-Fab-IDS (verenafusp alfa)	[42]
	Metachromatic leukodystrophy */Arylsulfatase A (ASA)	HIRMAb-ASA	[43]
	Sanfilippo A (MPSIIIA) */Sulfamidase (SGSH)	HIRMAb-SGSH	[44]
	Sanfilippo B (MPSIIIB) */*N*-acetyl-alpha-D-glucosaminidase (NAGLU)	HIRMAb-NAGLU	[45]
	Niemann-Pick A/B */Acid sphingomyelinase (ASM)	HIRMAb-ASM	[46]
	Tay-Sachs */Hexosaminidase A (HEXA)	HIRMAb-HEXA	[46]
	Batten Type 1 */Palmitoyl-protein thioesterase (PPT1)	HIRMAb-PPT1	[46]
	GM1-gangliosidosis */b-galactosidase (GLB1)	HIRMAb-GLB1	[46]
	Alzheimer’s */Anti-Aβ amyloid single-chain Fv antibody (scFv)	HIRMAb-Aβ bi-specific antibody	[47]
	Parkinson’s, Alzheimer’s, and/or stroke */Tumor Necrosis Factor Decoy Receptor (TNFR)	HIRMAb-TNFR	[48]
	Parkinson’s, Alzheimer’s, and/or stroke */Erythropoietin (EPO)	HIRMAb-EPO	[49]
	Parkinson’s and/or stroke */Glial cell-derived neurotrophic factor (GDNF)	HIRMAb-GDNF	[50]
	Stroke, neural repair */Brain-derived neurotrophic factor (BDNF)	HIRMAb-BDNF	[51]
	Organophosphate exposure/Paroxonase-1 (PON1)	HIRMAb-PON1	[52]
	Various/Any mono-biotinylated therapeutic	HIRMAb-avidin	[53]
Transferrin Receptor (TfR)	Hurler syndrome (MPS I)/IDUA	hTfR-Fab-IDUA (lepunafusp alfa)	[33]
	Hurler syndrome (MPS I)/IDUA	mTfRMAb-IDUA	[54]
	Hunter syndrome (MPS II)/IDS	hTfRMAb-IDS (pabinafusp alfa)	[34]
	Hunter syndrome (MPS II)/IDS	TfR transport vehicle, ETV:IDS (DNL310)(tividenofusp alfa)	[36]
	Hunter syndrome (MPS II)/IDS	mTfRMAb-IDS	[55]
	Sanfilippo A (MPSIIIA) */SGSH	hTfR-Fab-SGSH (postnafusp alfa)	[56]
	Sanfilippo A (MPSIIIA) */SGSH	TfR transport vehicle, ETV:SGSH (DNL126)	[57]
	Sanfilippo A (MPSIIIA) */SGSH	mTfRMAb-SGSH	[58]
	Sanfilippo B (MPSIIIB) */NAGLU	hTfR-Fab-NAGLU (JR-446)	[59]
	Pompe Disease/*α*-glucosidase (GAA)	TfR transport vehicle, ETV:GAA (DNL952)	[60]
	Alzheimer’s */Anti-Aβ amyloid MAb	TfR-scFab-Aβ-MAb (trontinemab)	[35]
	Alzheimer’s */Anti-Aβ amyloid scFv	mTfRMAb-Aβ bi-specific antibody	[61]
	Parkinson’s, Alzheimer’s, and/or stroke */TNFR	mTfRMAb-TNFR	[62]
	Parkinson’s, Alzheimer’s, and/or stroke */EPO	mTfRMAb-EPO	[63]
	Parkinson’s and/or stroke */GDNF	mTfRMAb-GDNF	[64]
	Various/Any mono-biotinylated therapeutic	mTfRMAb-avidin	[65]
	Alzheimer’s */Anti-Aβ amyloid MAb	TfR-tetravalent bispecific RmAb158-scFv8D3	[66]
Insulin-Like Growth Factor-1 Receptor (IGF1R)	Hunter syndrome (MPS II)/IDS	IGF1R3H5-IDS	[67]

^1^ The transport domain of the fusion proteins targeting the BBB human insulin receptor (HIR) is a complete MAb targeting the HIR (HIRMAb) or a Fab fragment (HIR-Fab-IDS). The transport domain of the fusion proteins targeting the BBB transferrin receptor (TfR) is a MAb targeting the human (h) TfR (hTfRMAb), a MAb targeting the mouse (m) TfR (mTfRMAb), a Fab fragment targeting the hTfR, or a TfR transport vehicle (ETV) targeting the hTfR. The transport domain in IGF1R3H5-IDS is a lama antibody targeting the human BBB insulin-like growth factor 1 receptor (IGF1R). * represents an indication that has a primary CNS disease burden.

**Table 2:** Completed and in-progress clinical trials with brain-penetrating fusion proteins targeting BBB receptors.

Indication	Therapeutic Domain	Fusion Protein	ID
Hurler syndrome (MPS I)	Iduronidase (IDUA)	HIRMAb-IDUA (valanafusp alpha, AGT-181)	Phase I/II completed NCT02371226, NCT02597114, NCT03053089, NCT03071341
Hurler syndrome (MPS I)	IDUA	hTfR-Fab-IDUA (lepunafusp alfa, JR-171)	Phase I/II in progress, NCT04227600, NCT04453085
Hunter syndrome (MPS II)	Iduronate-2-sulfatase (IDS)	HIRMAb-IDS (AGT-182)	Phase I completed NCT02262338
Hunter syndrome (MPS II)	IDS	hTfRMAb-IDS (pabinafusp alfa, JR-141)	Phase I/II completed, NCT03128593, NCT03359213 Phase III in progress, NCT03568175, NCT04573023 Approved in Japan
Hunter syndrome (MPS II)	IDS	TfR transport vehicle, ETV:IDS (tividenofusp alfa, DNL310)	Phase II/III in progress, NCT04251026, NCT05371613, NCT06075537 Approved by the FDA
Hunter syndrome (MPS II)	IDS	HIR-Fab-IDS (verenafusp alfa, GNR-055)	Phase I completed, phase II in progress, NCT05208281
Sanfilippo A (MPSIIIA)	Sulfamidase (SGSH)	hTfR-Fab-SGSH (postnafusp alfa, JR-441),	Phase I/II in progress, NCT06095388
Sanfilippo A (MPSIIIA)	SGSH	TfR transport vehicle, EVT:SGSH (DNL126)	Phase I/II in progress, NCT06181136
Sanfilippo B (MPSIIIB)	N-acetyl-alpha-D-glucosaminidase (NAGLU)	hTfR-Fab-NAGLU (JR-446)	Phase I/II in progress, NCT06488924
Pompe Disease	*α*-glucosidase (GAA)	TfR transport vehicle, ETV:GAA (DNL952)	Phase I in progress, NCT07354724
Alzheimer’s	Anti-Aβ amyloid MAb	TfR-scFab-Aβ-MAb (trontinemab)	Phase I/II in progress, NCT04639050, NCT07169578

**Table 3:** Somatic improvement in pediatric subjects with MPSI on valanafusp alpha (HIRMAb-IDUA) for 52 weeks [32].

Parameter	Change at 52 Weeks from Baseline *
Liver volume	Decreased 23% (*p* < 0.0005)
Spleen volume	Decreased 26% (*p* < 0.0005)
Shoulder flexion ** (w26)	Increased 10.9 degrees (*p* < 0.01)
Shoulder extension ** (w26)	Increased 9.5 degrees (*p* < 0.01)

* 9/11 patients were already in ERT before the study. ** Data computed at week 26.

## Data Availability

No new data were created or analyzed in this study. Data sharing is not applicable to this article.

## Abbreviations

The following abbreviations are used in this manuscript:

AD	Alzheimer’s disease
apoTf	iron-free transferrin
ARC	arcuate nucleus
ARIA	amyloid-related imaging abnormalities
ATV	antibody transport vehicle
ASA	arylsulfatase A
ASM	acid sphingomyelinase
ASO	antisense oligonucleotide
AUC	area under the curve
BBB	blood–brain barrier
BDNF	brain-derived neurotrophic factor
BEC	brain endothelial cell
BMEC	brain microvascular endothelial cell
BMV	brain microvessel
CHO	Chinese hamster ovary
CNS	central nervous system
CSF	cerebrospinal fluid
CVO	circumventricular organ
DS	dermatan sulphate
ECD	extracellular domain
EGF	epidermal growth factor
EGFR	EGF receptor
EPO	erythropoietin
Fab	fragment antigen-binding region
GAG	glycosaminoglycans
GDNF	glial-cell-derived neurotrophic factor
Gfap	glial fibrillary acidic protein
GLP	Good Laboratory Practice
GLU1	glucose transporter type 1
GNR-055	HIR-Fab-IDS
GUSB	glucuronidase
HEXA	hexosaminidase A
HIR	human insulin receptor
HIR-A	short isoform of HIR
HIR-B	long isoform of HIR
holoTf	diferric-loaded transferrin (holotransferrin)
HS	heparan sulfate
hTfR	human TfR
ID	injected dose
IDUA	iduronidase
IDS	iduronate-2-sulfatase
IGF-1	insulin-like growth factor
IGF1R	insulin-like growth factor-1 receptor
IP	intraperitoneal
IV	intravenous
JR-141	pabinafusp alfa
JR-171	lepunafusp alfa
JR-441	postnafusp alfa
JR-446	hTfRMAb-NAGLU fusion protein
*K_D_*	dissociation constant
LAT1	large neutral amino acid transporter type 1
LSDs	lysosomal storage disorders
MAb	monoclonal antibody
MLD	metachromatic leukodystrophy
MoA	mechanism of action
monoTf	monoferric transferrin
mTfR	mouse TfR
M6P	mannose-6-phosphate
NAGLU	*N*-acetyl-alpha-D-glucosaminidase
NEP	neprilysin
PD	Parkinson’s disease
PET	positron emission tomography
PI3K	phosphoinositide 3-kinase
PON1	paroxonase-1
PPT1	palmitoyl-protein thioesterase
PS	Permeability–surface area
RME	receptor-mediated endocytosis
RMT	receptor-mediated transcytosis
rTfR	rat TfR
RTK	receptor tyrosine kinase
scFv	single-chain Fv
sdAbs	single-domain antibodies
SGSH	sulfamidase
SPR	surface plasmon resonance
SQ	subcutaneous
SST	somatostatin
Tf	transferrin
TfR	transferrin receptor
TNF	tumor necrosis alfa
TNFR	TNF receptor
VD	volume of distribution
